# Brain Metabolic Features of *FUS*‐ALS: A 2‐[ ^18^F]FDG‐PET Study

**DOI:** 10.1002/ana.27201

**Published:** 2025-02-20

**Authors:** Antonio Canosa, Umberto Manera, Rosario Vasta, Grazia Zocco, Francesca Di Pede, Sara Cabras, Filippo De Mattei, Francesca Palumbo, Barbara Iazzolino, Emilio Minerva, Luca Sbaiz, Maura Brunetti, Salvatore Gallone, Maurizio Grassano, Enrico Matteoni, Giulia Polverari, Giuseppe Fuda, Federico Casale, Paolina Salamone, Giovanni De Marco, Giulia Marchese, Cristina Moglia, Andrea Calvo, Marco Pagani, Adriano Chiò

**Affiliations:** ^1^ ALS Center, ‘Rita Levi Montalcini’ Department of Neuroscience University of Turin Turin Italy; ^2^ Azienda Ospedaliero‐Universitaria Città della Salute e della Scienza di Torino, Neurology Unit 1U Turin Italy; ^3^ Institute of Cognitive Sciences and Technologies, CNR Rome Italy; ^4^ University of Camerino, Center for Neuroscience Camerino Italy; ^5^ Department of Clinical Pathology Azienda Ospedaliero‐Universitaria Città della Salute e della Scienza di Torino, Laboratory of Genetics Turin Italy; ^6^ Positron Emission Tomography Center AFFIDEA‐IRMET S.p.A Turin Italy; ^7^ Neuroscience Institute of Turin (NIT) Turin Italy; ^8^ Department of Medical Radiation Physics and Nuclear Medicine Karolinska University Hospital Stockholm Sweden

## Abstract

**Objective:**

We aimed at evaluating the brain metabolic features of fused in sarcoma amyotrophic lateral sclerosis (*FUS*‐ALS) compared with sporadic ALS (sALS), using 2‐[fluorine‐18] fluoro‐2‐deoxy‐D‐glucose positron emission tomography (2‐[^18^F]FDG‐PET).

**Methods:**

We employed the 2‐sample *t*‐test model of SPM12, implemented in MATLAB, to compare 12 *FUS*‐ALS cases with 40 healthy controls (HC) and 48 sALS, randomly collected from the series of patients who underwent brain 2‐[^18^F]FDG‐PET at the ALS Center of Turin (Italy) at diagnosis from 2009 to 2019. In the comparisons between cases and HC, we included age at PET and sex as covariates. Because *FUS*‐ALS usually shows early onset in spinal regions, in the comparison between *FUS*‐ALS and sALS, we included singularly the following covariates in a second step, to evaluate the determinants of eventual metabolic differences: age at PET, sex, and onset (spinal/bulbar).

**Results:**

sALS patients showed significant relative hypometabolism in bilateral fronto‐temporo‐occipital cortex and right insula as compared with *FUS*‐ALS. After adjusting for age, the relative hypometabolism remained in the bilateral precentral gyrus and in the right middle and inferior temporal gyrus. As compared with HC, *FUS* patients displayed a significant relative hypermetabolism in the pontobulbar region and right cerebellar tonsil, dentate nucleus, and uvula, while sALS showed relative hypometabolism in bilateral frontal and occipital cortices and in left temporal and parietal regions.

**Interpretation:**

Patients with *FUS*‐ALS show relative preservation of motor cortex metabolism compared with those with sALS, possibly reflecting the prevalence of lower motor neuron impairment in their phenotype. Prospective studies are necessary to investigate the possible role of 2‐[^18^F]FDG‐PET as a biomarker to track disease spreading in clinical trials. ANN NEUROL 2025;97:1134–1143

Amyotrophic lateral sclerosis (ALS) is a relentlessly progressive neurodegenerative disease affecting upper (UMN) and lower (LMN) motor neurons, causing wasting and weakness of voluntary muscles and leading to death within 2–5 years from onset, mainly due to respiratory failure.^1^ Approximately, 10% of ALS patients have a positive family history (familial ALS, fALS), while the remaining 90% of cases are apparently sporadic (sporadic ALS, sALS). The most common ALS‐related genes are superoxide dismutase 1 (*SOD1*), TAR DNA‐binding protein (*TARDBP*), fused in sarcoma (*FUS*), and chromosome 9 open reading frame 72 (*C9orf72*), which account for approximately 60% of fALS and 10% of sALS.^2^ Particularly, *FUS* mutations can be found in up to 3% of fALS and 0.3% of sALS in European countries, with higher frequency in Asian populations. Nevertheless, *FUS* is the most commonly mutated gene in juvenile cases (ie, onset <25 years).^3^ Currently, the relevance of identifying *FUS* mutations in persons with ALS has been increased by the scenario of a possible treatment. Indeed, the intrathecal administration of an antisense oligonucleotide (ASO) reducing the expression of the gene has been proved to reduce the burden of FUS aggregates in neurons, which are a pathological hallmark of disease in patients carrying *FUS* mutations (*FUS*‐ALS).^4^ Based on these premises, a phase 1–3, randomized, placebo‐controlled study to evaluate the efficacy, safety, pharmacokinetics, and pharmacodynamics of the mentioned anti‐*FUS* ASO in *FUS*‐ALS is currently ongoing in many ALS centers in America, Europe, and Asia (ClinicalTrials.gov Identifier: NCT04768972). In the view of making *FUS*‐ALS a treatable disease, the role of biomarkers is essential to track disease trajectories and collect data on the natural history of the neurodegenerative process. The added value of neuroimaging is the possibility to evaluate *in vivo* structural and functional changes due to the neurodegenerative process of ALS. Data from animal models of *FUS*‐ALS, including *Caenorhabditis elegans*, *Drosophila melanogaster*, and rodents, indicate a convergence of findings, which collectively point to damage to the neuromuscular junction.^5^ Moreover, neuropathological^6^ and clinical data^3^ indicate that the damage to LMN is more severe than that to UMN in cases of *FUS*‐ALS. When considered collectively, these premises highlight the potential value of brain imaging in evaluating the neurodegenerative process associated with ALS across its various subtypes. Currently, the literature lacks neuroimaging studies assessing structural and functional changes in *FUS*‐ALS, with the exception of case reports.^7^ Therefore, our aim was to investigate the brain metabolic features of *FUS*‐ALS using 2‐[fluorine‐18] fluoro‐2‐deoxy‐D‐glucose positron emission tomography (2‐[^18^F]FDG‐PET).

## Methods

We adhered to the relevant Strobe checklist.

### Study Participants

We retrospectively included 12 patients carrying *FUS* mutations, diagnosed with genetically determined ALS according to El Escorial revised diagnostic criteria^8^ at the ALS Center of Turin (‘Rita Levi Montalcini’ Department of Neuroscience, University of Turin, Turin, Italy), between 2008 and 15 March 2024. A comparison group of patients diagnosed with definite, probable and probable laboratory‐supported sALS according to the revised El Escorial diagnostic criteria,^8^ without mutations in the major ALS‐related genes (ie, *SOD1*, *TARDBP*, *FUS*, and *C9ORF72*) was considered. From the sample of patients who underwent brain 2‐[^18^F]FDG‐PET at the time of diagnosis between 2009 and 2019 at our center, we randomly collected 4 patients per *FUS* subject, totaling 48 sALS patients. For all ALS patients demographic and clinical features, including age and disease duration at PET, sex, site of onset (spinal/bulbar), cognitive status (normal/altered) at PET, and King's stage at PET were collected. Details about the neuropsychological assessment can be found elsewhere.^9^ The King's stage was calculated from the ALSFRS‐R score according to a published algorithm.^10^

We also included in the analyses 40 healthy controls (HC). We considered eligible as controls subjects referred to the PET Center for suspected lung cancer (1) with no oncologic disease detected, (2) with brain PET scan reported as normal by the nuclear medicine physician, (3) without history of neurological disorders, and (4) with normal neurological examination.

### Genetic Analysis

All patients underwent genetic analysis for *C9ORF72*, *SOD1*, *TARDBP*, and *FUS* genes. All the coding exons and 50 bp of the flanking intron‐exon boundaries of *SOD1*, *TARDBP*, and *FUS* have been polymerase chain reaction (PCR) amplified, sequenced using the BigDye Terminator v3.1 sequencing kit (Applied Biosystems), and run on an ABIPrism 3,500 genetic analyzer. A repeat‐primed PCR assay was used to screen for the presence of the GGGGCC hexanucleotide expansion in the first intron of *C9ORF72*. A cutoff of ≥30 repeats was considered pathological.^11^

### 2‐[ ^18^F]FDG‐PET Image Acquisition and Pre‐processing

Brain 2‐[^18^F]FDG‐PET was performed according to published guidelines.^12^ Patients fasted at least 6 hours before the exam. Blood glucose was <7.2 mmol/l in all cases before the procedure. After a 20‐minutes rest, 185–200 MBq of 2‐[^18^F]FDG was injected. The acquisition started 60 minutes after the injection. PET/computed tomography (CT) scans were performed on a Discovery ST‐E System (General Electric). Brain CT and PET scan were sequentially acquired, the former being used for attenuation correction of PET data. The PET images were reconstructed with 4 iterations and 28 subsets with an initial voxel size of 2.34 × 2.34 × 2.00 mm, and data were collected in 128 × 128 matrices. Images were spatially normalized to a customized brain 2‐[^18^F]FDG‐PET template^13^ and subsequently smoothed with a 10‐mm filter in MATLAB R2018b (MathWorks, Natick, MA, USA). Intensity normalization was performed at individual level averaging each voxel for the mean value of the whole brain.

### Statistical Analysis

The demographic and clinical characteristics of *FUS*, sALS patients and HC were compared using the *χ* ^2^‐test for categorical variables and the Mann–Whitney test for quantitative, continuous variables. We used the full factorial design as implemented in SPM12 to test the hypothesis that differences among groups (*FUS*, sALS, and HC) exist overall (ie, main effect of groups). Age at PET and sex were used as covariates, and the height threshold was set at *p* < 0.001 (*p* < 0.05 family‐wise error [FWE]‐corrected at cluster level).

In case the hypothesis was confirmed, comparisons among groups were performed through the 2‐sample *t*‐test model of SPM12. In the comparisons between cases and HC, we included age at PET and sex as covariates. As *FUS*‐ALS usually shows early onset of symptoms in spinal regions,^3^ and in view of the limited size of the *FUS*‐ALS group, in the comparison between *FUS*‐ALS and sALS, we included singularly the following covariates in a second step of the analyses, to evaluate the determinants of eventual metabolic differences: age at PET, sex, and onset (spinal/bulbar). In all group comparisons, the height threshold was set at *p* < 0.001 (*p* < 0.05 FWE‐corrected at cluster level). In case of absence of significant differences, we adopted the *p* < 0.005 height threshold (*p* < 0.05 FWE‐corrected at cluster level).

In all the analyses, only clusters containing >125 contiguous voxels were considered significant. Brodmann areas (BAs) were identified at a 0–2‐mm range from the Talairach coordinates of the SPM output isocenters corrected by Talairach Client (http://www.talairach.org/index.html ).

### Standard Protocol Approvals, Registrations, and Patient Consents

All participants signed a written informed consent, and the study was approved by the ethical committee of the ‘Azienda Ospedaliero‐Universitaria Città della Salute e della Scienza di Torino’.

## Results

### Demographic and Clinical Data

Demographic and clinical characteristics of patients and HC are reported in Table 1. Age at PET of *FUS*‐ALS subjects resulted significantly lower as compared with sALS and HC. Moreover, sALS and *FUS*‐ALS patients significantly differed in terms of site of onset, being bulbar onset absent in the *FUS*‐ALS group. Among *FUS* patients, the following mutations were identified: p.Asn63Ser, p.Arg495X, p.Lys510Arg (2 subjects), p.Arg514Gly, p.Arg514Ser (2 subjects), p.Arg521Cys, p.Arg521Gly (2 subjects), and p.Arg521His (2 subjects). None of the *FUS*‐ALS patients had tremor, epilepsy, learning disability, mental retardation, or other developmental disorders, which have been reported in some patients carrying *FUS* mutations.^3^, ^14^ All of them were of Caucasian ancestry. In Supplementary Table, we summarize the *FUS* variants identified in our series together with the familial status of the carriers, the consequences on protein sequence, the affected protein domain, and the classification of pathogenicity according to the ClinVar database. No patients carry *C9ORF72* expansion. Only 1 case carries a *SOD1* variant (c.59A > G, p.Asn20Ser): he has familial ALS segregating with the *FUS* p.Arg514Ser variant, while the *SOD1* variant is classified as a variant of unknown significance and does not segregate with the disease in the kindred. Moreover, all the cases in this pedigree show the same phenotype including early onset, axial and proximal upper limb weakness, soon followed by respiratory failure, which is one of the typical presentations of *FUS*‐ALS.^15^ As regards the case with cognitive impairment, he carries the p.Arg521Cys pathogenic variant, and no developmental disorder was reported in the past medical history.

**TABLE 1:** Comparison of Demographic and Clinical Characteristics of sALS, *FUS*‐ALS Patients, and Healthy Controls (HC)

Parameter	sALS (N = 48)	*FUS*‐ALS (N = 12)	HC (N = 40)	
	Median (IQR)	Median (IQR)	Median (IQR)	*p*‐Value^a^
Age at FDG‐PET (years)^c^	65.5 (56.3–70.5)	47.0 (41.0–52.7)	66.5 (55.0–72.0)	**<0.001**
Duration at FDG‐PET (months)	12.9 (9.1–20.2)	24.5 (12.8–29.2)	‐	**0.021**
Total ALSFRS‐r score at FDG‐PET	43.0 (38.0–45.0)	39.0 (34.5–41.7)	‐	**0.014**

	n (%)	n (%)		*p* ^b^
Sex				0.826
Female	16 (33.3)	4 (33.3)	11 (27.5)	
Male	32 (66.7)	8 (66.7)	29 (72.5)	
Site of onset				**0.042**
Bulbar onset	13 (27.1)	0 (0.0)	‐	
Spinal onset	35 (72.9)	12 (100.0)	‐	
Cognitive status				0.205
CN	24 (50.0)	9 (75.0)	‐	
ALSbi	5 (10.4)	0 (0.0)	‐	
ALSci	6 (12.5)	0 (0.0)	‐	
ALScbi	1 (2.1)	1 (8.3)	‐	
Missing	12 (25.0)	2 (16.7)	‐	
Cognitive status				0.147
Cognitively normal	24 (50.0)	9 (75.0)	‐	
Cognitively altered	12 (25.0)	1 (8.3)	‐	
Missing	12 (25.0)	2 (16.7)	‐	
King's staging system				
Stage 1	22 (45.8)	5 (41.7)	‐	0.912
Stage 2	13 (27.1)	4 (33.3)	‐	
Stage 3	13 (27.1)	3 (25.0)	‐	

Significant differences are reported in bold.

^a^ Kruskal–Wallis test.

^b^ Chi‐squared test.

^c^ Mann–Whitney *U* test with Bonferroni correction resulted significant only in pairwise comparison between *FUS*‐ALS and sALS and *FUS*‐ALS and controls (*p* = 0.002).

### 2‐[ ^18^F]FDG‐PET Results

#### Full factorial analysis

The full factorial design resulted in a significant main effect of groups (*p* < 0.001; data not shown). We hence computed the *post‐hoc* comparisons among the 3 groups.

#### FUS‐ALS *vs* sALS

**FIGURE 1:**
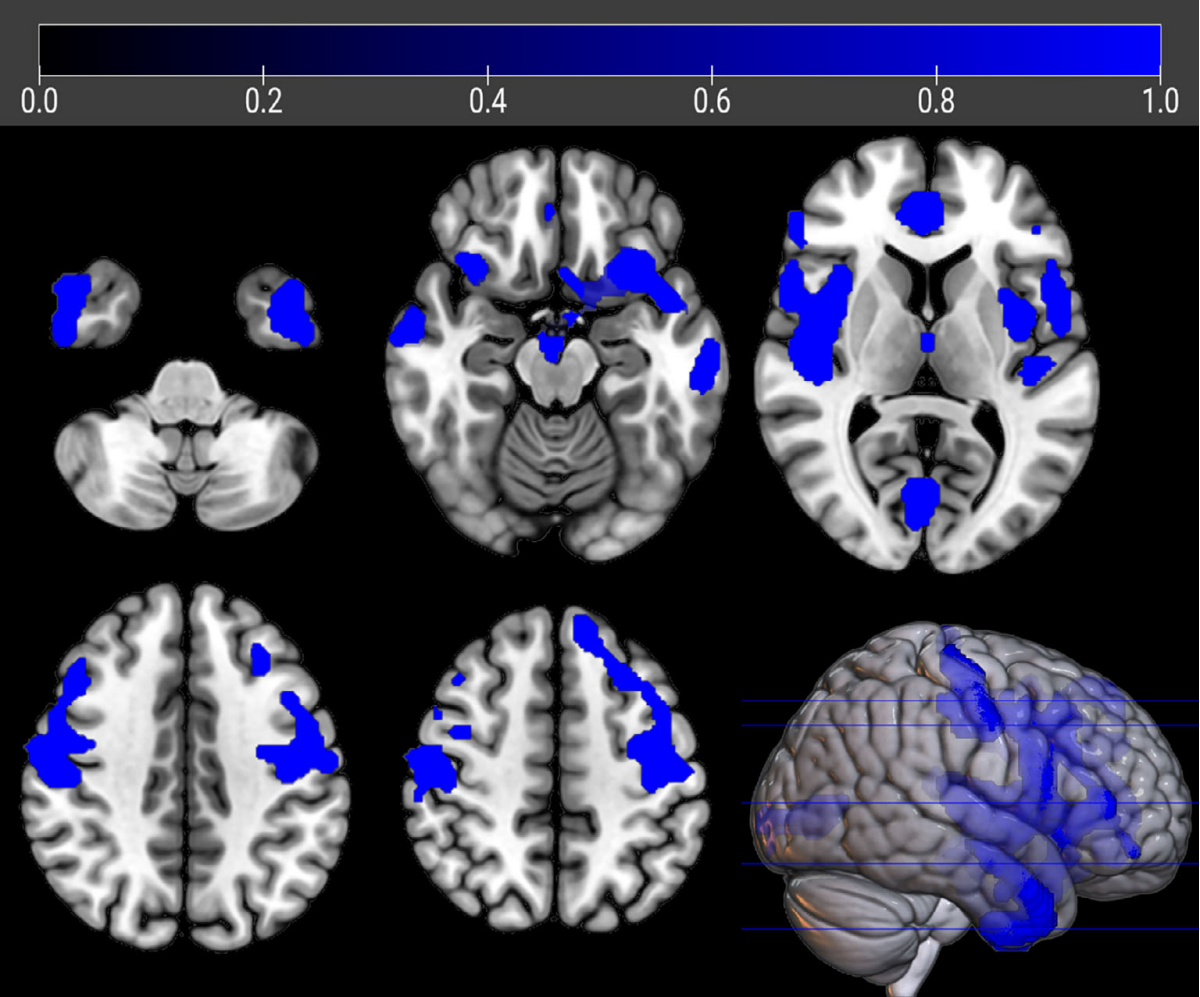
The regions showing a statistically significant relative hypometabolism in sALS patients as compared to *FUS*‐ALS subjects are marked in blue and are reported on axial sections of a brain MRI template and on the brain surface of a glass brain rendering (bottom right).

**FIGURE 2:**
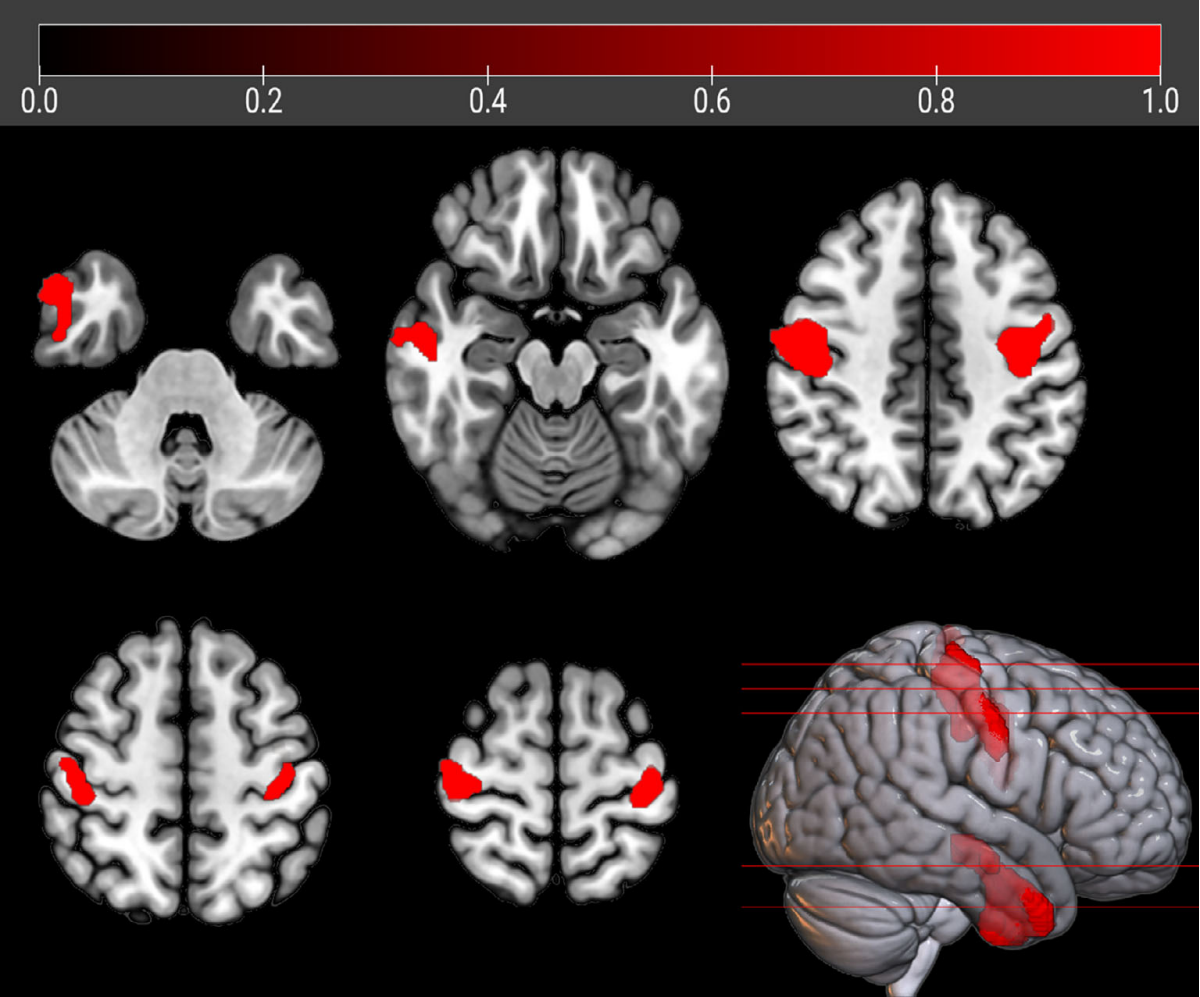
The regions showing a statistically significant relative hypometabolism in sALS patients as compared to *FUS*‐ALS subjects after adjusting for age at PET are marked in red and are reported on axial sections of a brain MRI template and on the brain surface of a glass brain rendering (bottom right).

sALS patients showed significant relative hypometabolism in bilateral fronto‐temporo‐occipital cortex and right insula as compared with *FUS* patients (*p* < 0.001; Table 2, Fig 1). The results did not change when the analysis was adjusted separately for sex and onset site. (Supplementary Figs S1 and S2). However, after adjusting for age, the relative hypometabolism only remained in the bilateral precentral gyrus and in the right middle and inferior temporal gyrus (*p* < 0.005; Table 3, Fig 2).

**TABLE 2:** Clusters of Significant Relative Hypometabolism of sALS Patients as Compared with *FUS* Patients

*p*‐Value (FWE‐corrected)	Cluster Extent	Z‐Score	Talairach Coordinates (*x*, *y*, *z*)			Lobe	Region	BA
0.000	8,749	5.02	−42.0	−4.0	−40.0	Left temporal lobe	Inferior temporal gyrus	20
		4.92	−24.0	13.0	−17.0	Left frontal lobe	Inferior frontal gyrus	47
		4.85	−38.0	−6.0	44.0	Left frontal lobe	Middle frontal gyrus	6
0.000	6,738	4.65	40.0	2.0	5.0	Right insula	Insula	13
		4.64	53.0	4.0	0.0	Right temporal lobe	Superior temporal gyrus	22
		4.60	53.0	9.0	22.0	Right frontal lobe	Inferior frontal gyrus	44
0.003	828	4.52	−2.0	−74.0	6.0	Left occipital lobe	Cuneus	18
		3.65	6.0	−99.0	7.0	Right occipital lobe	Cuneus	18
		3.20	−10.0	−97.0	−5.0	Left occipital lobe	Lingual gyrus	18
0.000	1,184	3.56	10.0	30.0	24.0	Right limbic lobe	Anterior cingulate	32

BA = Brodmann area.

**TABLE 3:** Clusters of Significant Relative Hypometabolism of sALS Patients as Compared with *FUS* Patients after Adjusting for Age at PET

*p*‐Value (FWE‐corrected)	Cluster Extent	*Z*‐Score	Talairach Coordinates (*x*, *y*, *z*)			Lobe	Region	BA
0.016	1,356	3.84	−38.0	−7.0	45.0	Left frontal lobe	Precentral gyrus	6
		3.81	−28.0	−22.0	69.0	Left frontal lobe	Precentral gyrus	4
0.028	1,204	3.75	50.0	−8.0	−37.0	Right temporal lobe	Inferior temporal gyrus	20
		3.72	51.0	−3.0	−22.0	Right temporal lobe	Middle temporal gyrus	21
0.022	1,264	3.53	51.0	−4.0	39.0	Right frontal lobe	Precentral gyrus	6
		3.49	46.0	−17.0	41.0	Right frontal lobe	Precentral gyrus	4

BA = Brodmann area.

No clusters of significant relative hypermetabolism of sALS compared with *FUS* patients were found in any analyses.

#### FUS‐ALS *vs* HC

**FIGURE 3:**
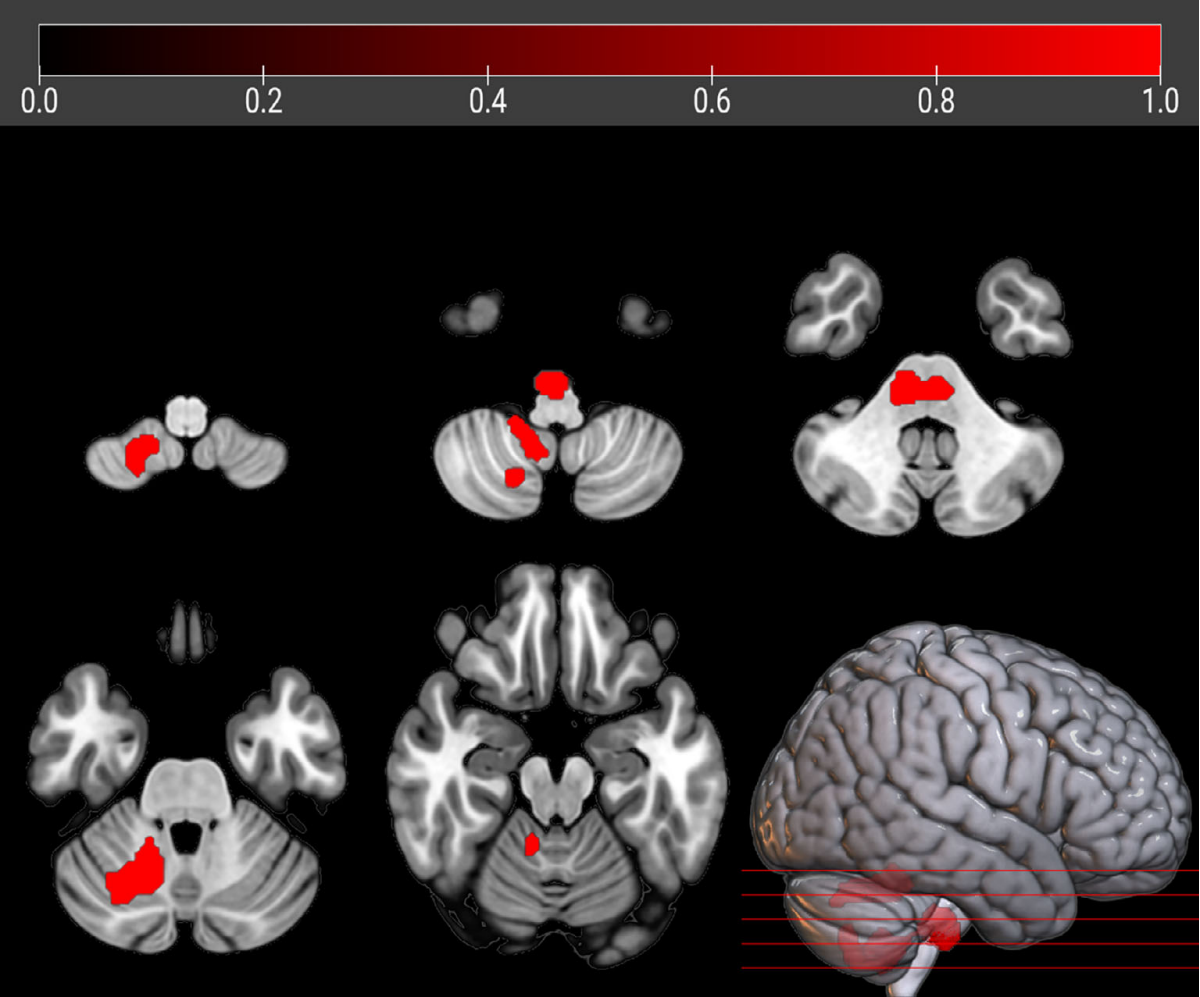
The regions showing a statistically significant relative hypermetabolism in *FUS*‐ALS patients as compared to HC after adjusting for age at PET and sex are marked in red and are reported on axial sections of a brain Magnetic Resonance Imaging template and on the brain surface of a glass brain rendering (bottom right).

*FUS* patients displayed a significant relative hypermetabolism in the pontobulbar region and right cerebellar tonsil, dentate nucleus, and uvula (*p* < 0.001; Fig 3). No clusters of significant relative hypometabolism of *FUS* patients were found.

#### sALS *vs* HC

We found a significant relative hypometabolism in sALS subjects compared with HC in bilateral frontal and occipital cortices and in left temporal and parietal regions (*p* < 0.001; Supplementary Fig S3 and Table S2). No clusters of significant relative hypermetabolism of sALS patients were found.

## Discussion

The key finding of the present study is that brain metabolism of *FUS*‐ALS is significantly different from sALS, and that age partially mediates this difference. The effect of age should be considered, because *FUS*‐ALS is typically associated with earlier onset as compared with sALS.^3^ Otherwise, sex and site of onset do not significantly impact, although spinal onset was significantly more frequent among *FUS*‐ALS, as previously reported.^3^ Indeed, after adjusting for age at onset of symptoms, we found a relative hypometabolism in sALS as compared with *FUS*‐ALS involving bilateral precentral gyrus and right middle and inferior temporal gyrus, suggesting a relative preservation of metabolism of these regions in *FUS*‐ALS.

A possible explanation lies in the clinical and neuropathological evidence that in *FUS*‐ALS LMN is more extensively affected as compared with UMN. In a single‐center series of *FUS* patients, we recently reported that almost 20% of cases showed a pure LMN phenotype, mainly with a flail leg variant.^3^ Neuropathological data point out that FUS‐immunoreactive cytoplasmic inclusions can be detected in a large number in LMN, while in the primary motor cortex they seem more frequent in the early onset cases.^6^ The interpretation of our results in the context of the spread of *FUS*‐related neurodegeneration is challenging. A number of studies on *FUS* mouse models^16^, ^17^ highlighted that neuromuscular junction disruption is an early event in pathogenesis of motor neuron degeneration, thus supporting a dying‐back mechanism starting from LMN in *FUS*‐ALS pathogenesis. An international study is currently underway to shed light on the natural history of ALS patients carrying *FUS* mutations (NH00004, A Retrospective Chart Review Study of the Natural History and Disease Progression in Amyotrophic Lateral Sclerosis Patients with Fused in Sarcoma Mutations, *FUS*‐ALS). Awaiting these data, some published studies addressed the regional spreading pattern in ALS as a whole, in terms of UMN and LMN clinical involvement. A single‐center retrospective study on a cohort of 913 Italian ALS patients suggested that subjects with predominant LMN impairment show more often a horizontal spreading from 1 side of the spinal cord to the other and that horizontal spreading is associated with proximal limb onset.^18^ A European multicenter collaboration enrolled 1,376 consecutively studied patients for whom information about UMN and LMN at onset and along the disease course was collected. According to the authors, LMN degeneration mostly progresses by contiguity, while UMN disease leads to an acceleration of rostral‐caudal LMN loss.^19^ Although not focused on *FUS* patients, these studies seem to provide some hints to hypothesize disease spreading patterns in this rare condition. Interestingly, in our cohort, 11 of 12 *FUS* patients displayed disease onset with LMN impairment and subsequent horizontal spreading of symptoms from one limb to the contralateral.

As regards to right temporal relative hypometabolism in sALS compared with *FUS*‐ALS, we can hypothesize a role of mild differences in cognitive function. Despite the lack of significant differences between the 2 groups in terms of cognitive status, a trend is detectable, with 75% of *FUS* patients compared with 50% of sALS displaying cognitive function sparing. This finding is in keeping with literature data pointing out that cognitive impairment seems to be rare in *FUS* patients and mainly detectable in young patients in the form of a mild cognitive impairment and learning disability.^3^

As regards to the comparisons with HC, sALS patients displayed metabolic changes that have been consistently reported before.^20^, ^21^ The interpretation of the relative hypermetabolism of *FUS* patients compared with HC in cerebellar and brainstem clusters can be only speculative. A recent article reported the association between brainstem hypermetabolism and shorter survival in ALS,^22^ providing a possible explanation for our finding. Indeed, *FUS*‐ALS is usually associated with a poor prognosis. The presence of FUS‐immunoreactive neuronal cytoplasmic inclusions in cerebellar dentate nucleus has been described in neuropathological studies as a characteristic of cases with early onset and fast progression.^6^ Nevertheless, how this finding can lead to metabolic changes remains unclear.

Neuroimaging has the goal of identifying neurodegenerative changes in vivo and might be complementary to clinical assessments for patient's classification and stratification in clinical trials.^23^ In this context, the possible role of 2‐[^18^F]FDG‐PET as a biomarker of disease spreading is worthy of in‐depth study, since the discovery of an anti‐*FUS* ASO^4^ is making the perspective of treating these patients realistic. In the ongoing phase 1–3 clinical trial on the anti‐*FUS* ASO, brain magnetic resonance imaging (MRI) has been incorporated into the screening process and is conducted annually to assess brain atrophy as an exploratory endpoint. In this view, PET imaging can enrich the information provided by structural MRI, disclosing early metabolic changes which precede grey matter loss.^24^ It is noteworthy that brain glucose metabolism, as assessed through 2‐[^18^F]FDG‐PET, has been already included as an outcome measure in clinical trials on patients with Alzheimer's disease.^25^ In the context of ALS, a potential role for 2‐[^18^F]FDG‐PET is also reasonable in clinical trials on presymptomatic subjects, given that it can disclose brain metabolic changes even before the onset of symptoms and the elevation of neurofilaments in *C9ORF72* expansion carriers.^26^ Moreover, it can be performed also in subjects who are not able to undergo MRI due to claustrophobia, severe respiratory failure, or presence of non‐compatible metals.

Of the mutations we identified in our series, all but 3 were listed as pathogenic in ClinVar. The classification of p.Asn63Ser was considered controversial. Therefore, we performed the analyses without the case carrying this mutation and the results were unchanged (data not shown). The p.Lys510Arg and the p.Arg514Ser were not included in ClinVar. The p.Lys510Arg variant was reported as pathogenic in a previous publication.^27^ The carriers in our series show a phenotype similar to that described in the original report: onset at 46 and 52 years of age, respectively, predominant lower motor neuron involvement, slow progression. One of the carriers of the p.Arg514Ser variant was reported in a previous article showing its segregation with the disease.^15^ The other carrier is her son. They both show a proximal onset at upper limbs.

The *FUS*‐ALS patients showed a longer disease duration at PET and a lower ALSFRS‐R score, even though the King's stage was not significantly different between groups. These differences, stress the concept that, even after a longer time from onset and a higher degree of disability, *FUS* patients still have a relative preservation of motor cortex metabolism as compared with sALS.

The current study follows on from previous studies from our center that have investigated the characteristics of brain metabolic changes associated with mutations in key ALS‐related genes. Comparing patients carrying the *C9ORF72* expansion with ALS patients who do not carry genetic mutations, with and without frontotemporal dementia, we found more widespread metabolic changes in *C9ORF72* patients, mainly relative hypometabolism in the frontotemporal cortex, in agreement with the evidence of an association of this gene with frontotemporal lobar degeneration.^28^ In a study focusing on ALS patients with *TARDBP* mutations,^29^ we found a relative hypometabolism of *TARDBP*‐ALS in the right pre‐ and postcentral gyrus, superior and middle temporal gyrus, and the insula, compared with patients without genetic mutations. The relative hypometabolism in regions giving rise to the corticospinal tracts seems in agreement with the predominantly pyramidal phenotype shown in these patients in population‐based studies.^30^ Otherwise, in our dataset, *SOD1*‐ALS was characterized by a relative hypermetabolism in the motor cortex as compared with ALS without genetic mutations and HC.^31^ The difference from non‐mutated ALS could be related to the most common phenotype of *SOD1*‐ALS, with a predominant lower motor neuron, flail leg picture. The relative hypermetabolism of the motor cortex in *SOD1* patients as compared with HC might be due to microglial activation, that has been shown in a previous PET study with the ^11^C‐PK11195 tracer.^32^ Interpretation of these findings in a common framework with the present study suggests that 2‐[^18^F]FDG‐PET is a good candidate biomarker to assess the extent of neurodegeneration in ALS in motor and extra‐motor regions and to establish correlations with phenotype.

This study is not without its flaws. First, the number of *FUS* patients is limited. However, *FUS* patients are rarely encountered and usually display a short survival, which hampers neuroimaging studies with adequate sample size. Accordingly, this is the largest series of *FUS* patients undergoing brain imaging so far. Second, conclusive evidence about the correlation between phenotypic features and brain metabolic changes in *FUS* patients cannot be drawn from our results, and larger prospective studies are needed. Third, we cannot rule out the possibility of a survival bias, since early onset *FUS* patients with short survival might be underrepresented in our sample.

In conclusion, our main finding is a relative preservation of motor cortex metabolism in *FUS* patients, compared with sALS subjects, which seems to reflect the prevalence of LMN impairment in their clinical phenotype. Our study supports the possible role of 2‐[^18^F]FDG‐PET as a biomarker to track disease spreading in clinical trials, and we warrant prospective studies to investigate this issue.

## Author Contributions

Conception and design of the study: A.Can., U.M., R.V., C.M., A.Cal., M.P., and A.Ch.

Acquisition and analysis of data: A.Can., U.M., R.V., G.Z., F.D.P., S.C., F.D.M., F.P., B.I., E.Mi., L.S., M.B., S.G., M.G., E.Ma., G.P., G.F., F.C., P.S., G.D.M., G.M., C.M., A.Cal., M.P., and A.Ch.

Drafting a significant portion of the manuscript or figures: A.Can., U.M., R.V., C.M., A.Cal., M.P., and A.Ch.

## Potential Conflicts of Interest

Nothing relevant to the current research.

## Supporting information


**Data S1.** Supporting Information.

## Data Availability

The NIfTI files of the clusters identified in the analyses will be available on demand by interested researchers.
