# Supplementary material for: Brain Metabolic Features of FUS‐ALS: A 2‐[ 18F]FDG‐PET Study
Source: Ann Neurol. 2025 Feb 20;97(6):1134–43. doi: 10.1002/ana.27201 (PMC12081991; doi:10.1002/ana.27201)

**Supplementary Table 1**. *FUS* variants identified in our series together with the consequences on protein sequence, the affected protein domain, and the classification of pathogenicity according to the ClinVar database. NLS: Nuclear Localization Signal. SYQG: Serine, Tyrosine, Glutamine, Glycine. FALS: Familial ALS. SALS: Sporadic ALS. F: Female. M: Male.

| **Mutation** | **Carriers** | **Consequences on protein sequence** | **Affected protein domain** | **ClinVar classification** |
| --- | --- | --- | --- | --- |
| p.Asn63Ser | 1 SALS patient (M) | Missense | SYQG-rich domain | Conflicting classifications |
| p.Arg495X | 1 SALS patient | Nonsense | Affecting NLS | Pathogenic |
| p.Lys510Arg | 2 FALS patients (1 M, 1 F) | Missense | NLS | Not listed |
| p.Arg514Gly | 1 FALS patient (M) | Missense | NLS | Pathogenic |
| p.Arg514Ser | 2 FALS patients (1 F, 1 M) | Missense | NLS | Not listed |
| p.Arg521Cys | 1 SALS *de novo* patient (M) | Missense | NLS | Pathogenic |
| p.Arg521Gly | 2 FALS patients (2 M) | Missense | NLS | Pathogenic |
| p.Arg521His | 1 SALS (M) and 1 FALS (F) patients | Missense | NLS | Pathogenic |

**Supplementary Table 2**. Clusters of significant relative hypometabolism of sALS patients as compared to HC (BA=Brodmann Area).

| p(FWE-corrected) | Cluter extent | Z-score | Talairach coordinates (x, y, z) | | | Lobe | Region | BA |
| --- | --- | --- | --- | --- | --- | --- | --- | --- |
| 0.000 | 9965 | 5.84 | -50.0 | 0.0 | 41.0 | Left Frontal Lobe | Middle Frontal Gyrus | 6 |
|  |  | 5.19 | -46.0 | 33.0 | 4.0 | Left Frontal Lobe | Inferior Frontal Gyrus | 45 |
|  |  | 5.04 | -38.0 | 18.0 | 45.0 | Left Frontal Lobe | Middle Frontal Gyrus | 8 |
| 0.000 | 6109 | 5.01 | -36.0 | -76.0 | -5.0 | Left Occipital Lobe | Inferior Occipital Gyrus | 19 |
|  |  | 4.91 | -6.0 | -77.0 | 6.0 | Left Occipital Lobe | Lingual Gyrus | 18 |
|  |  | 4.67 | 6.0 | -79.0 | 9.0 | Right Occipital Lobe | Cuneus | 17 |
| 0.000 | 5193 | 4.69 | 44.0 | 4.0 | 42.0 | Right Frontal Lobe | Middle Frontal Gyrus | 6 |
|  |  | 4.41 | 50.0 | 33.0 | -2.0 | Right Frontal Lobe | Inferior Frontal Gyrus | 47 |
|  |  | 4.19 | 46.0 | -9.0 | 52.0 | Right Frontal Lobe | Precentral Gyrus | 4 |
| 0.000 | 1631 | 4.47 | -61.0 | -11.0 | -20.0 | Left Temporal Lobe | Inferior Temporal Gyrus | 20 |
|  |  | 4.30 | -63.0 | -32.0 | -15.0 | Left Temporal Lobe | Middle Temporal Gyrus | 21 |
| 0.004 | 794 | 4.16 | -53.0 | -58.0 | 36.0 | Left Parietal Lobe | Inferior Parietal Lobule | 40 |
|  |  | 3.84 | -40.0 | -67.0 | 20.0 | Left Temporal Lobe | Middle Temporal Gyrus | 39 |
| 0.014 | 597 | 3.89 | 24.0 | 39.0 | -27.0 | Right Frontal Lobe | Orbital Gyrus | 11 |
|  |  | 3.72 | 10.0 | 40.0 | -19.0 | Right Frontal Lobe | Inferior Frontal Gyrus | 11 |
|  |  | 3.63 | -6.0 | 36.0 | 11.0 | Left Limbic Lobe | Anterior Cingulate | 32 |

**Supplementary Figure 1.** The regions showing a statistically significant relative hypometabolism in sALS patients as compared to *FUS*-ALS subjects after adjusting for sex are marked in blue and are reported on axial sections of a brain Magnetic Resonance Imaging template and on the brain surface of a glass brain rendering (bottom right)


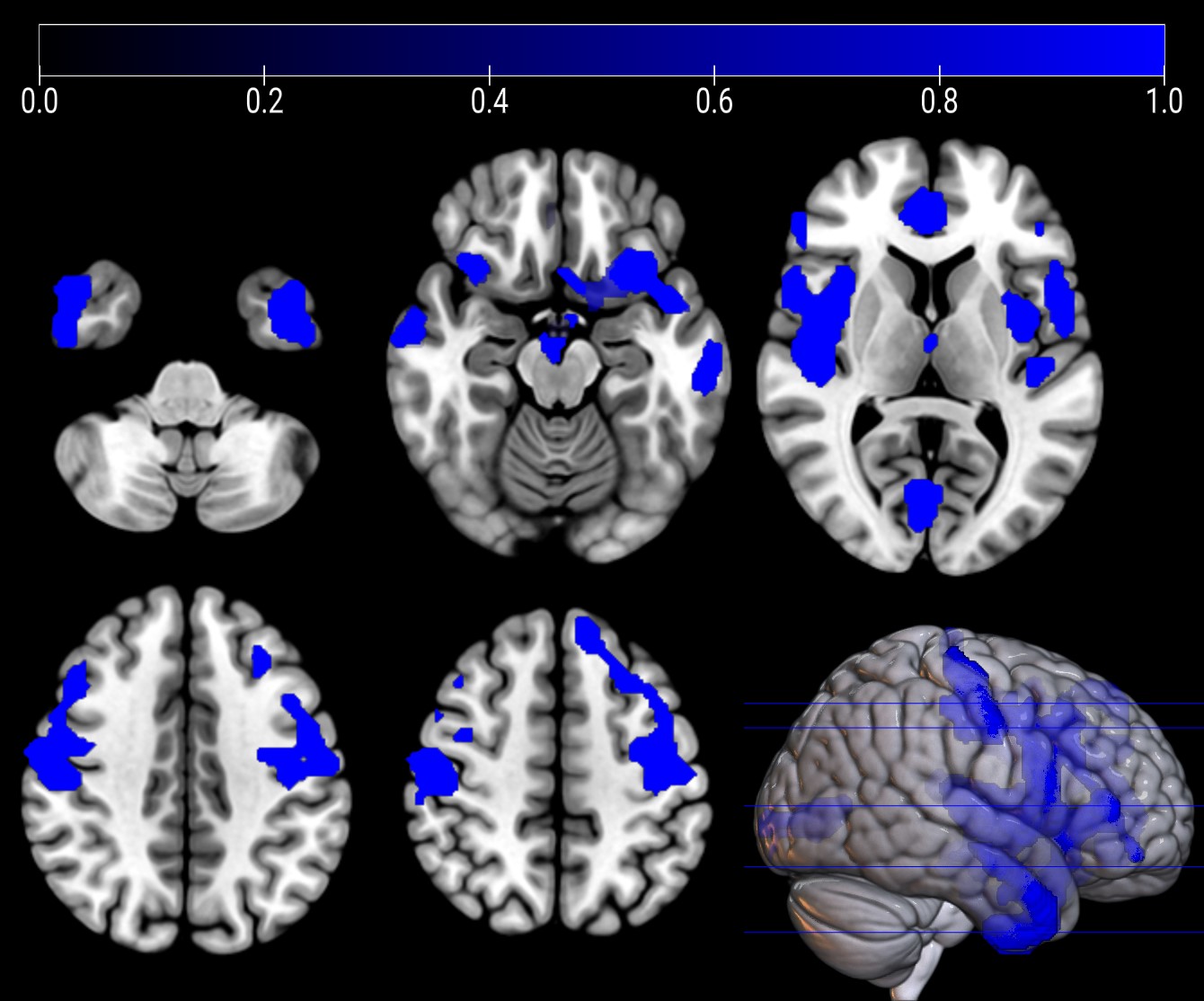


**Supplementary Figure 2.** The regions showing a statistically significant relative hypometabolism in sALS patients as compared to *FUS*-ALS subjects after adjusting for site of onset are marked in blue and are reported on axial sections of a brain Magnetic Resonance Imaging template and on the brain surface of a glass brain rendering (bottom right)


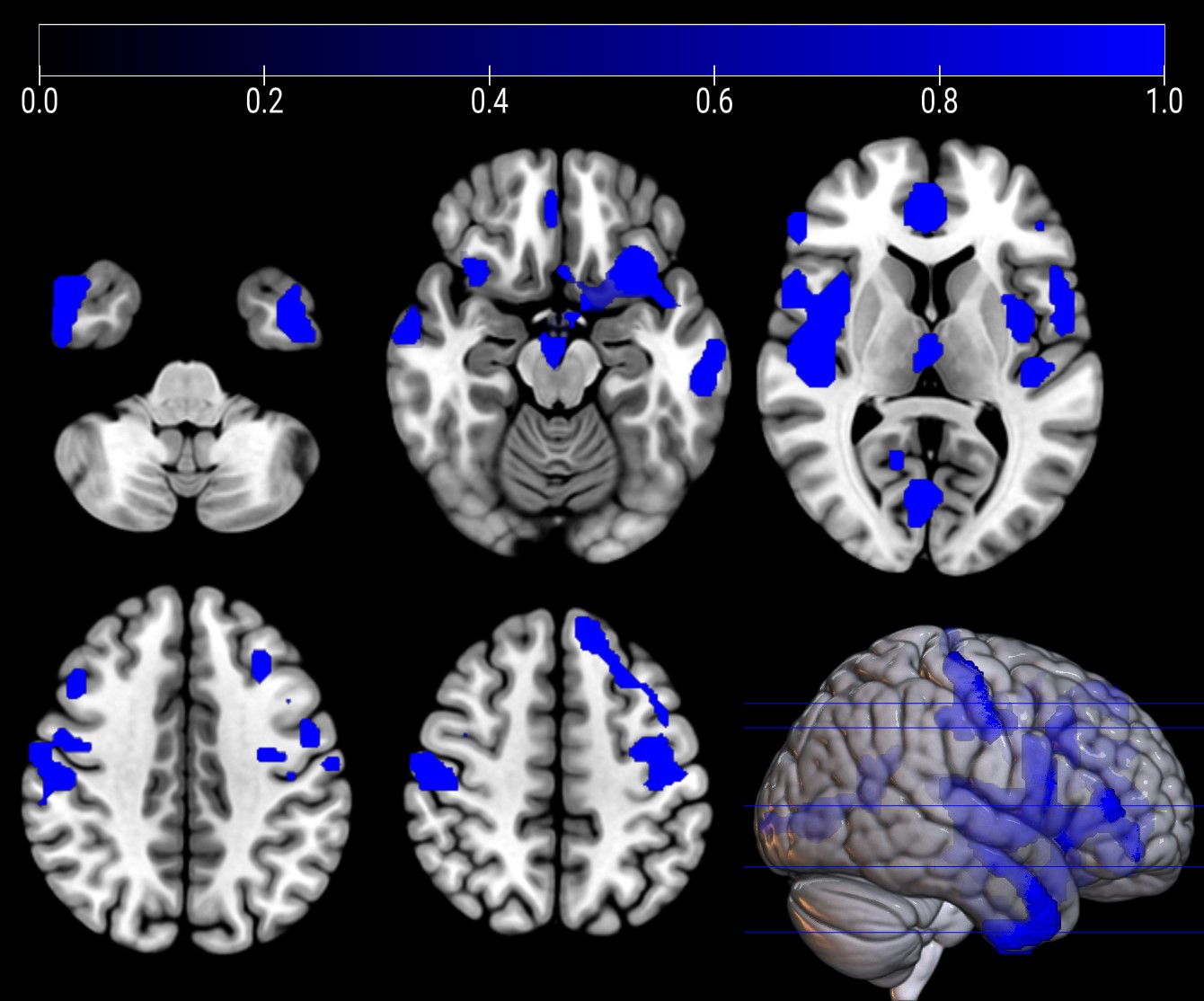


**Supplementary Figure 3.** The regions showing a statistically significant relative hypometabolism in sALS patients as compared to HC after adjusting for age at PET and sex are marked in red and are reported on axial sections of a brain Magnetic Resonance Imaging template and on the brain surface of a glass brain rendering (bottom right)


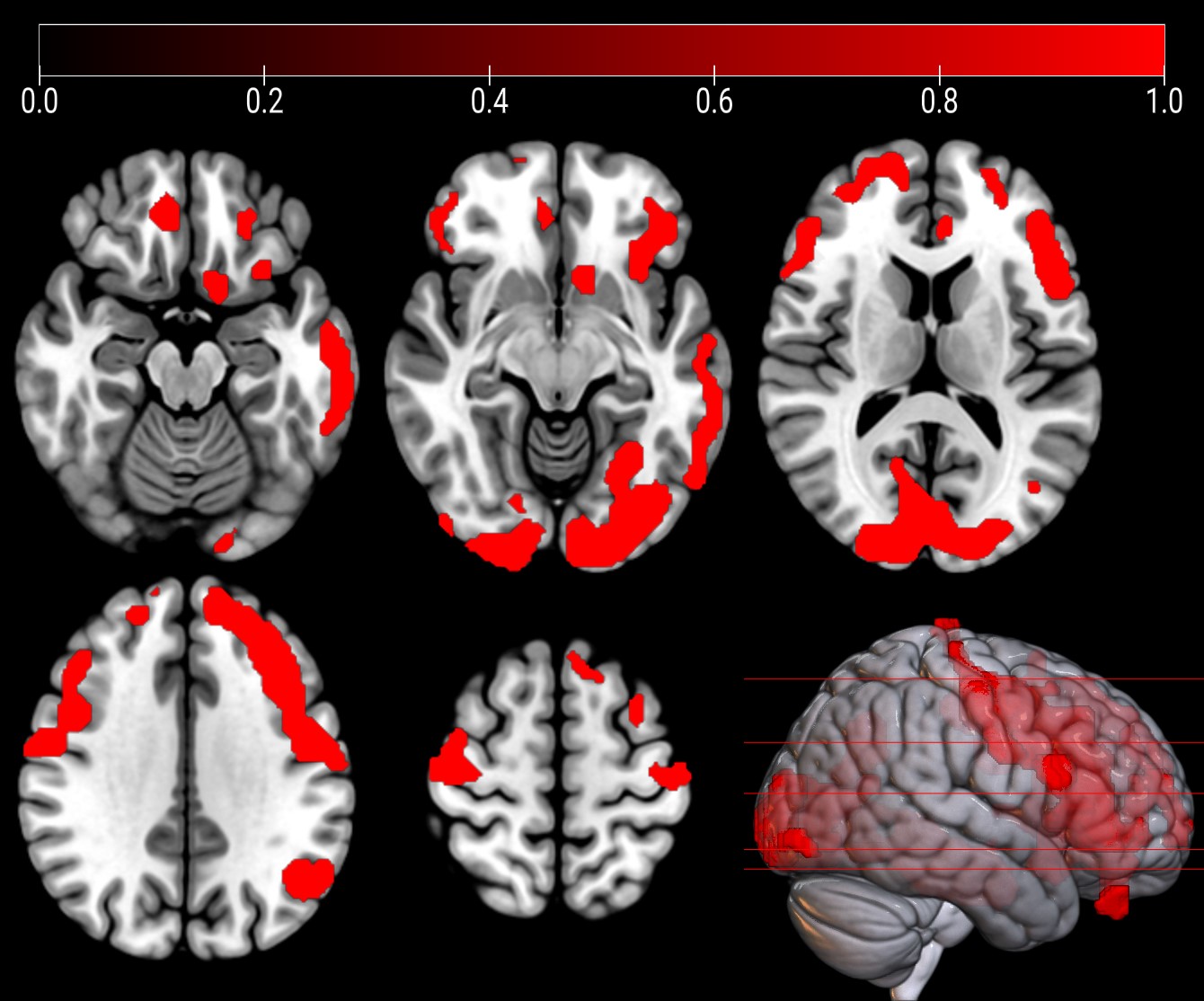

Supplement: Supplementary file 1 — Data S1. Supporting Information. [file ANA-97-1134-s001.doc]
